# Ultrasound-Sensitive Liposomal Exatecan for Tumor-Specific Drug Release for Treatment of Pancreatic Cancer

**DOI:** 10.3390/pharmaceutics18010003

**Published:** 2025-12-19

**Authors:** Dongyeon Kim, Hyungwon Moon, Sunyoung Han, Hak Jong Lee

**Affiliations:** 1 Department of Applied Bioengineering, Graduate School of Convergence Science and Technology, Seoul National University, 1 Gwanak-ro, Gwanak-gu, Seoul 08826, Republic of Korea; dongyeonk619@snu.ac.kr; 2 R&D Center, IMGT Co., Ltd., 172 Dolma-ro, Bundang-gu, Seongnam-si 13605, Gyeonggi-do, Republic of Korea; hyungwon.moon@nanoimgt.com (H.M.); sunyoung.han@nanoimgt.com (S.H.); 3 Department of Radiology, Seoul National University Bundang Hospital, 82 Gumi-ro 173, Bundang-gu, Seongnam-si 13620, Gyeonggi-do, Republic of Korea

**Keywords:** exatecan mesylate, ultrasound-sensitive liposome, focused ultrasound, pancreatic cancer, nanomedicine, tumor-targeted chemotherapy

## Abstract

**Background:** Pancreatic ductal adenocarcinoma (PDAC) remains one of the most lethal cancers, largely due to its dense stromal architecture and poor intratumoral drug penetration. To address this challenge, IMP305 was developed as an ultrasound-sensitive liposome for tumor-localized drug release. In particular, IMP305 is dominantly capable of release by ultrasound-mediated cavitation. **Methods:** This ultrasound-sensitive liposome integrates tumor-specific drug delivery with cavitation-induced loosening of the stromal architecture in PDAC, thereby enabling more efficient intratumoral drug release using PANC-1 xenografted mouse. **Results:** The maximal tolerance dose of exatecan was increased by encapsulation into IMP305. Cavitation-triggered structural disruption of IMP305 was 84.68 ± 6.21%, which resulted in a robust release of approximately 84.4 ± 1.95% of the encapsulated exatecan. In PANC-1 xenograft models, IMP305 exhibited a maximal tolerance dose approximately four times higher than that of free exatecan and demonstrated markedly superior antitumor ability. Especially, IMP305 combined with focused ultrasound achieved the most pronounced therapeutic benefit, demonstrating a 49.17 ± 9.00% reduction in tumor volume at day 48 and an 80% survival rate at day 60. **Conclusions:** In conclusion, these findings demonstrate that ultrasound-activated IMP305 significantly enhances intratumoral accumulation and release of exatecan, resulting in superior tumor suppression while mitigating systemic toxicity.

## 1. Introduction

Pancreatic ductal adenocarcinoma (PDAC) remains one of the most lethal malignancies, with a five-year survival rate below 12% [1]. The failure of systemic chemotherapy in PDAC is largely attributable to its unique tumor microenvironment (TME), which is characterized by excessive stromal fibrosis, low vascular density, and high interstitial fluid pressure [2,3]. These features severely restrict perfusion and drug permeability, leading to heterogeneous and subtherapeutic intratumoral drug concentrations even under dose-intense regimens. Consequently, conventional cytotoxic drugs such as gemcitabine and FOLFIRINOX provide only marginal improvement in patient survival [4]. Dose escalation for more accumulation in the tumor is restricted by intolerable adverse effects.

Since conventional chemotherapeutic agents are rarely delivered to PDAC, highly potent chemotherapeutics are needed for effective PDAC suppression. Among cytotoxic agents, topoisomerase I (Topo-I) inhibitors represent one of the most potent classes, inducing irreversible DNA damage and apoptosis in proliferating tumor cells. Exatecan mesylate (DX-8951f), a next-generation topoisomerase I (Topo-I) inhibitor, is fully active without metabolic conversion and exhibits higher Topo-I inhibition than SN-38 [5,6]. Nevertheless, clinical trials with free exatecan were terminated due to dose-limiting myelosuppression and gastrointestinal toxicity, emphasizing the need for precise spatiotemporal control of such potent agents [7].

Recently, antibody–drug conjugates (ADCs) have emerged as another breakthrough strategy for selective chemotherapy and reduction of adverse effects. By covalently linking highly potent payloads to tumor-specific antibodies via cleavable linkers, ADCs achieve targeted drug activation at the cellular level [8]. The recent success of trastuzumab deruxtecan (Enhertu^®^) and datopotamab deruxtecan in breast and lung cancers highlights the clinical potential of Topo-I-based ADCs using exatecan or deruxtecan payloads [9,10]. These platforms demonstrate the therapeutic value of combining a stable linker system with a diffusible, membrane-permeable payload that exerts a “bystander effect” in neighboring cells [11,12]. Nevertheless, ADCs’ efficacy fundamentally depends on the density and uniformity of antigen expression and on efficient internalization conditions that are rarely satisfied in PDAC [13]. Furthermore, the large molecular size of ADCs (~150 kDa) limits penetration through the thick stromal matrix of PDAC, leaving antigen-negative or hypoperfused regions untreated [14].

These challenges underscore the need for a physically triggered, antigen-independent delivery strategy capable of circumventing the profound biological heterogeneity of PDAC [15]. Focused ultrasound (FUS) enables non-invasive deposition of mechanical acoustic forces with high three-dimensional precision at deep tumor sites, providing a controllable strategy for modulating the tumor microenvironment [16]. Under non-thermal acoustic conditions, FUS can induce cavitation events—oscillation and collapse of gas bubbles—that transiently disrupt cellular membranes and remodel the extracellular matrix [17,18]. The mechanical stresses generated by cavitation, including microstreaming and shear forces, have been shown to loosen stromal architecture, reduce interstitial resistance, and facilitate deeper penetration of therapeutics into desmoplastic tumors. In our previous study, cavitation-mediated mechanical forces enhanced drug transport in a controlled and non-destructive manner, without causing thermal injury to surrounding tissues [19]. Compared to the thermal effect, mechanical cavitation dominantly effected only penetration of drugs to the PDAC without thermal burn and systemic toxicity. This feature is particularly important in PDAC, where heating can damage adjacent digestive organs or precipitate acute and mild pancreatitis [20,21].

Given the mechanical modulatory capabilities of FUS, integrating focused ultrasound with liposomal drug carriers represents a promising strategy for achieving spatiotemporally controlled chemotherapy. However, most FUS-sensitive liposomal systems are thermosensitive liposomes for selective release. Thermosensitive formulations rely on localized hyperthermia to trigger payload release [22,23]. Despite its utility in specific applications, heat-mediated activation poses the potential for thermal damage. These limitations highlight the need for an alternative, non-thermal FUS-triggered mechanism capable of modulating the stromal barrier and enabling deeper intratumoral drug delivery [24].

**Figure 1:**
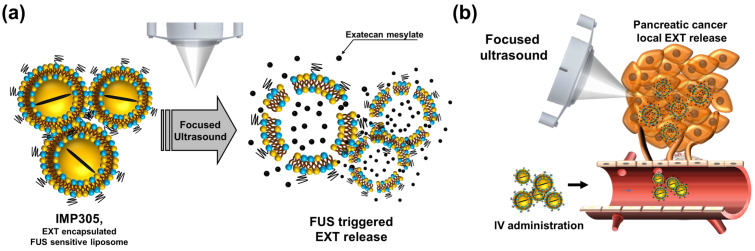
Schematic illustration of (**a**) mode of action of EXT in IMP305 and (**b**) tumor-specific release and accumulation by FUS-mediated cavitation.

Here, in order to enable deeper penetration into PDAC through mechanical forces and simultaneously reduce adverse effects, we developed an ultrasound-sensitive liposomal formulation of exatecan mesylate, IMP305 (Figure 1a). This formulation was designed to combine the benefits of liposomal encapsulation for reduction of systemic toxicity and tumor-specific EXT release by ultrasound exposure (Figure 1a). Particularly, IMP305 exhibits a dominant exatecan release triggered by ultrasound-mediated cavitation rather than thermal mechanisms. This characteristic of IMP305 is anticipated to enhance penetration across the stromal barriers surrounding the tumor (Figure 1b).

In this study, we investigated the physicochemical properties of IMP305, optimized the EXT loading conditions, and evaluated the responsiveness to FUS irradiation by measurement of the EXT release ratio. Furthermore, we investigated in vitro cellular uptake and cytotoxicity in PANC-1 pancreatic cancer cells, as well as the in vivo safety, biodistribution, and antitumor efficacy of IMP305 in a xenograft mouse model. Our findings demonstrate that IMP305 provides a promising strategy for precise and tumor-targeted chemotherapy of PDAC, potentially overcoming the clinical limitations of free exatecan mesyltate.

## 2. Materials and Methods

### 2.1. Materials

1,2-Distearoyl-sn-glycero-3-phosphocholine (DSPC, purity; 99.9%), N-(carbonyl-methoxypolyethyleneglycol-2000)-1,2-distearoyl-sn-glycero-3-phosphoethanolamine sodium salt (DSPE-mPEG2000, purity; 99.6%), and 1,2-dioleoyl-sn-glycero-3-phosphoethanolamine (DOPE, purity; 99.9%) were purchased from Lipoid AG (Steinhausen, Switzerland). 1-Stearoyl-2-lyso-sn-glycero-3-phosphocholine (MSPC; also referred to as S-LysoPC, purity; 99.4%) was obtained from NOF Corporation (Tokyo, Japan). Exatecan mesylate (EXT, purity; >99.0%) was obtained from Chemieliva Pharmaceutical (Chongqing, China). Cholesterol (purity; 99.6%), ammonium sulfate, hydrochloric acid, sodium hydroxide, MTS assay kit (CellTiter 96^®^, Promega, Annandale, NSW, Australia), and L-histidine were obtained from Sigma-Aldrich (St. Louis, MO, USA). Sucrose was purchased from CheilJedang (Seoul, Republic of Korea).

### 2.2. Preparation of EXT-Loaded Liposomes (IMP305)

The phospholipid weight ratio of IMP305 was DSPC 9.4%, DSPE-mPEG 16.6%, cholesterol 13.8%, DOPE 57.1%, and MSPC 3.1%. IMP305 was prepared via the ethanol injection method. The detailed procedure is as follows: first, the DSPC-1.13 g, DSPE-mPEG 1.99 g, cholesterol 1.65 g, DOPE 6.85 g, and MSPC 0.37 g were dissolved in ethanol at a total lipid concentration of 256 mg/mL by stirring at 50 °C for 1 h. Subsequently, the lipid mixture was added dropwise to the 250 mM ammonium sulfate at the final lipid concentration of 16 mg/mL under stirring at 500 rpm. The ethanol content in the final aqueous formulation was 5.84% (*v*/*v*). This multilamellar vesicle (MLV) was size-extruded through polycarbonate filters using a LIPEX™ ThermoBarrel Extruder (Evonik Transferra Nanosciences, Burnaby, BC, Canada). The MLVs were initially extruded five times at 110 psi using N_2_ gas through a 200 nm pore-size polycarbonate membrane and sequentially extruded over 15 times through an 80 nm pore-size polycarbonate membrane at 250 psi. The temperature during size extrusion was maintained at 50 °C using a water circulator connected to the ThermoBarrel of the LIPEX system. After size extrusion, the ammonium sulfate was changed to 10% sucrose and 10 mM histidine buffer at pH 5.0 by dialysis (MWCO, 3 kDa). The buffer exchange was completed by measurement of conductivity.

The encapsulation of EXT was subsequently performed with the drug-to-lipid ratio of 1:4. After mixing at 50 °C for 2 h, removal of free EXT and buffer exchange to a pH 6.5 buffer were conducted using a HiPrep™ 26/10 Desalting column on the ÄKTA go system. Finally, IMP305 with the final EXT concentration of 2 mg/mL was diluted by 10% sucrose and 10 mM histidine buffer and IMP305 was sterilized by a 0.2 µm pore-size cellulose acetate membrane filter.

### 2.3. Analysis of Size Distribution, Morphology, and Encapsulation Efficiency of IMP305

The size distribution and zeta potential of IMP305 were measured by dynamic light scattering (DLS; Nano ZS90, Malvern Panalytical, Malvern, UK). The size distribution and zeta potential were measured three times with 50 times dilution using 10% sucrose and 10mM histidine buffer (pH 6.5). And the encapsulation efficiency of EXT was determined using a UV–visible spectrophotometer after removing unencapsulated EXT with a PD-10 desalting column. The concentration of EXT was quantified by absorbance at 370 nm. Additionally, the morphology of the IMP305 was visualized by cryo-transmission electron microscopy (cryo-TEM). For cryo-TEM analysis, samples were prepared using a Vitrobot-type plunge freezer (FEI FP5350/60). A 5 μL aliquot of the liposome suspension was applied to a carbon-coated TEM grid (Lacey support film, 200 mesh; Ted Pella, Inc., Redding, CA, USA), blotted with filter paper, and rapidly vitrified by plunging into liquid ethane. The quantification of membrane disruption by ultrasound was measured by visual observation of size increase and disappearance of EXT in cryo-TEM images (*N* = 10).

### 2.4. Optimization of Ion-Exchanged EXT Encapsulation Efficiency of IMP305

The encapsulation efficiency of EXT was determined after removal of unencapsulated drug using a size extrusion PD-10 column. The EXT concentration in IMP305 was quantified by UV absorbance at 370 nm. Encapsulation efficiency (%) was calculated as follows.

$$Encapsulation\;efficiency\;\left(\%\right)=\frac{Encapsulated\;drug}{Total\;drug\;added}\times 100$$

Optimization of EXT encapsulation was conducted by varying the type of ionic compound, the drug-to-lipid ratio, and the incubation temperature and time. To assess the impact of ionic species on encapsulation efficiency, empty liposomes were prepared using ammonium sulfate, ammonium phosphate, ammonium acetate, or ammonium citrate at a concentration of 250 mM. For evaluation of the optimal drug-to-lipid ratio, the lipid concentration was fixed at 16 mg/mL, while the drug amount was varied to achieve weight ratios of 1:8, 1:4, and 1:3. Additionally, to determine the most favorable encapsulation conditions, formulations were incubated at 25 °C, 40 °C, and 60 °C for 30 or 60 min, and the resulting encapsulation efficiencies were compared.

### 2.5. In Vitro Release Analysis of IMP305 Under Ultrasound Exposure

To evaluate cavitation-triggered EXT release from IMP305, 2 mL of IMP305 (0.5 mg/mL) was exposed to continuous-wave ultrasound using a UP200S equipped with a Sonotrode S3 (Hielscher Ultrasonics, Teltow, Germany). For generation of ultrasound-mediated cavitation, an ultrasound wave at low frequency (24 kHz, non-focal) was applied. The ultrasonicator tip was positioned at the center of the suspension, and ultrasound was applied at 92 W/cm^2^ for 30, 60, 120, or 180 s. Following sonication, the released EXT was purified and quantified by optical absorbance at 370 nm using an ÄKTA Go system. The release profile was also compared with that of a non-ultrasound-sensitive liposome composed of DSPC and DSPE-mPEG2k under the same ultrasound conditions.

For the in vitro release study under conditions mimicking the physiological environment including ultrasound treatment, IMP305 containing EXT was placed in a dialysis membrane (MWCO 3.0 kDa) and incubated in DMEM supplemented with 10% FBS. Focused ultrasound (FUS) was applied at 0.5 and 3 h using 2.0 kW/cm^2^, a 1% duty cycle, a pulse repetition frequency of 250 Hz, and a 10 s exposure per spot, consistent with the parameters for treatment of PDAC. The released EXT in the medium was collected via solid-phase extraction and quantified by HPLC (Luna 3 µm Phenyl-Hexyl 100 Å, 4.6 × 100 mm; methanol/dichloromethane gradient; 0.5 mL/min; 55 °C; detection at 254 nm).

### 2.6. In Vitro Cellular Uptake and Cytotoxicity of IMP305

Human pancreatic cancer cells (PANC-1) were used to evaluate cellular uptake and cytotoxicity. PANC-1 cells (ATCC, Manassas, VA, USA) were cultured in DMEM supplemented with 10% fetal bovine serum and 1% penicillin–streptomycin at 37 °C in a 5% CO_2_ incubator. For cellular uptake analysis, cells were seeded at 2 × 10^4^ cells/well in an 8-well chamber slide and incubated overnight. IMP305 encapsulating dextran sulfate–FITC for fluorescence imaging was then added to each well. Prior to treatment, ultrasound irradiation was applied using an ultrasonicator (24 kHz, 92 W/cm^2^, 20% amplitude, cycle 1, 1 min). After treatment of IMP305, cells were washed with PBS, fixed with 4% paraformaldehyde, and counterstained with Hoechst 33342. Cellular uptake was visualized using a confocal laser scanning microscope (LSM 710, Carl Zeiss, Oberkochen, Germany). Using ImageJ software (version 1.8.0, National Institutes of Health, Bethesda, ME, USA), the mean fluorescence intensity of the cells was measured by analyzing images for quantitative comparison of uptake efficiency.

The cytotoxicity of IMP305 and free EXT was assessed using an MTS assay (CellTiter 96^®^, Promega, Annandale, NSW, Australia). PANC-1 cells were seeded in 96-well plates at a density of 5 × 10^3^ cells/well. Free EXT or IMP305 at the concentrations of 25, 10, 5, 2, 1, and 0.5 nM was applied to the cells with or without ultrasound exposure (24 kHz, 92 W/cm^2^, amplitude 20%, 1 min). IMP305 and free EXT were pre-exposed to ultrasound prior to administration to PANC-1 cells. Seventy-two hours after treatment with IMP305 or free EXT, 20 μL of MTS solution was added to each well and incubated for 2 h at 37 °C. Absorbance was measured at 490 nm using a microplate reader (Infinite^®^ 200, Tecan, Männedorf, Switzerland).

### 2.7. Temperature Simulation Regarding FUS Condition

Temperature was simulated for the estimation of its increase during the FUS treatment. In the simulation algorithm, the Rayleigh–Sommerfeld diffraction integral and angular spectrum approach were used to calculate the acoustic field generated by the FUS transducer [25,26]. The resulting field was used to compute the temperature distribution by solving the bio-heat transfer equation [27]. The FUS transducer is a 4-channel concave annular array with a focal length of 6.1 cm and an aperture radius of 8 cm. The propagation medium consisted of 5.1 cm of water and 1 cm of soft tissue.

For each channel, the one-dimensional complex pressure along the radial axis perpendicular to the beam axis was obtained using the Rayleigh–Sommerfeld integral, and the phased-array pressure field was computed by superposing these pressures with phase adjustments for electronic beam steering. The two-dimensional acoustic field was generated by propagating this pressure distribution from z = 0 to 9 cm at 0.02 cm intervals using the angular spectrum method and subsequently interpolated into a three-dimensional Cartesian field for heat-generation calculations. The bio-heat transfer equation was solved using a finite-difference time-domain scheme based on the computed acoustic field. As the treatment position shifted, the focal point of the 3D acoustic field was aligned accordingly, and heat deposition was recalculated. The resulting 3D temperature distribution was obtained for each sonication, and temperature–time curves at each focus and the 2D peak temperature distribution at the focal depth of 6.1 cm were extracted.

### 2.8. Tumor-Specific Release of IMP305 Under the FUS Irradiation

In this study, all animal experiments were conducted in accordance with the Institutional Animal Care and Use Committee (IACUC No. BA-2411-404-005-02, date of approval: 27 December 2024) of Seoul National University Bundang Hospital. To investigate FUS-triggered EXT release, FUS irradiation was performed using an IMD10 system (IMGT Co., Ltd., Seongnam-si, Republic of Korea). The FUS transducer had an aperture of 88 mm, a radius of curvature of 61 mm, and operated at 1.1 MHz. At the −6 dB level, the focal region exhibited a cigar-shaped profile with a diameter of 1.2 mm, a length of 9.4 mm, and a focal depth of 38 mm. A 128-element linear array imaging probe (IMG B; 7.0 MHz center frequency; 40 mm length) was centrally mounted within the transducer aperture for US image guidance. The water bath was degassed and maintained at 37 °C prior to treatment. PANC-1 xenografted mice were anesthetized with a zoletil/rompun mixture (1:1:8 in saline). The following FUS parameters were used: intensity—2.5 kW/cm^2^, duty cycle—2%, pulse repetition frequency (PRF)—250 Hz, and exposure duration—20 s per spot. Tumors in the IMP305 + FUS group were irradiated at 1 h post-injection to maximize tumor accumulation prior to drug release.

For the fluorescent analysis, aluminum phthalocyanine tetrasulfonate (ex/em; 670/680 nm) as an alternative to EXT was encapsulated into IMP305 until saturation. The fabrication protocol was the same as the method in Section 2.2. The aluminum pthalocyanine and empty liposome were mixed at a 1:1 mass ratio for the saturated encapsulation. The unencapsulated aluminum pthalocyanine was removed by size exclusion chromatography.

After intravenous administration of aluminum-pthalocyanine-loaded IMP305, the whole bodies of mice were visualized by an in vivo imaging system (IVIS) before and after FUS irradiation. The fluorescent intensity of the tumor region was quantified as radiant efficiency using Living Image software version 4.8.2 (PerkinElmer, Waltham, MA, USA). The region of interest was automatically defined to cover the entire tumor region, and the average of radiant efficiency was integrated to determine fluorescence intensity. Quantitative intensity was demonstrated as average ± standard deviation (SD), and statistical analysis was performed using repeated-measures ANOVA with significance set at *p* < 0.05.

### 2.9. Comparison of Systemic Toxicity in IMP305 and Free EXT

To evaluate systemic toxicity of IMP305 and free EXT, male ICR mice (6 weeks old) were used to monitor the change of body weight. Briefly, IMP305 was intravenously administered at doses of 20 and 40 mg/kg for EXT in IMP305, and 3, 6, 9, and 12.5 mg/kg were intravenously administered for free EXT. Each formulation was administered 4 times on days 1, 5, 8, and 12. And the body weight was measured twice a week. Five mice in each group were used for the statistical analysis.

### 2.10. Antitumor Efficacy of IMP305 with the Combination of FUS

PANC-1 xenografted mice were applied for the enhancement of antitumor efficacy of IMP305 under the FUS irradiation. Male BALB/c nude mice (7 weeks old, Orient Bio, Seongnam-si, Gyeonggi-do, Republic of Korea) were subcutaneously inoculated with a 5 × 10^6^ PANC-1 cell suspension in 150 μL of PBS/Matrigel (2:1) into the right flank. Upon reaching a tumor volume of approximately 100–200 mm^3^, mice were randomized into treatment groups: (i) control (vehicle), (ii) free EXT (9 mg/kg), (iii) free EXT + FUS, (iv) IMP305 (40 mg/kg, EXT), and (v) IMP305 + FUS (40 mg/kg, EXT). IMP305 and free EXT were intravenously administered twice per week for 2 weeks. After the administration of 1h, the tumor was exposed to the FUS with parameters of intensity—2.5 kW/cm^2^, duty cycle—2%, pulse repetition frequency (PRF)—250 Hz, and exposure duration—20 s per spot. Tumor volume was measured every 3–4 days with digital calipers and calculated using the formula. Body weight and survival were recorded throughout the study.

$$Tumor\;volume\left({mm}^{3}\right)=\frac{length\times {width}^{2}}{2}$$

### 2.11. Statistical Analysis

All data are expressed as the mean ± standard deviation (SD). Statistical comparisons between groups were performed using the Mann–Whitney test with GraphPad Prism software (version 9.3.1). A *p*-value < 0.05 was considered statistically significant (*p* < 0.05).

## 3. Results and Discussion

### 3.1. The Characteristics of IMP305

**Figure 2:**
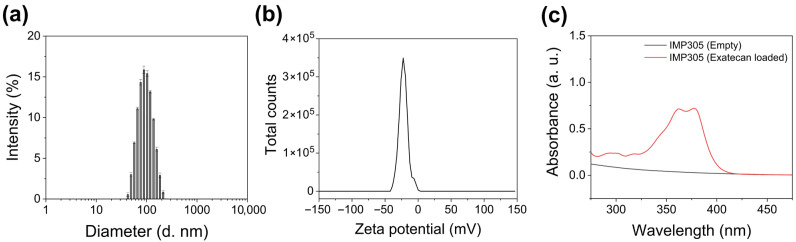
Characterization of IMP305. (**a**) Size distribution of IMP305. (**b**) Zeta potential of IMP305. (**c**) Absorbance spectrum of empty IMP305 (black line) and exatecan-loaded IMP305 (red line).

The ultrasound-sensitive liposomes encapsulating EXT, IMP305, were synthesized with the ion exchange mechanism for the EXT encapsulation. The size distribution of IMP305 was 88.5 ± 12.5 nm with a low polydispersity index (PDI) of 0.096 (Figure 2a). The zeta potential was measured to be −24.8 ± 12.5 mV, suggesting that the liposomes maintained a stable, negatively charged dispersion in aqueous solution (Figure 2b). UV–visible spectroscopy confirmed the successful encapsulation of EXT, as evidenced by the characteristic absorbance peak at 370nm of the EXT observed in the liposomal formulation (Figure 2c).

### 3.2. EXT Encapsulation Efficiency According to Ion Species, Drug-to-Lipid Ratio, and Temperature

**Figure 3:**
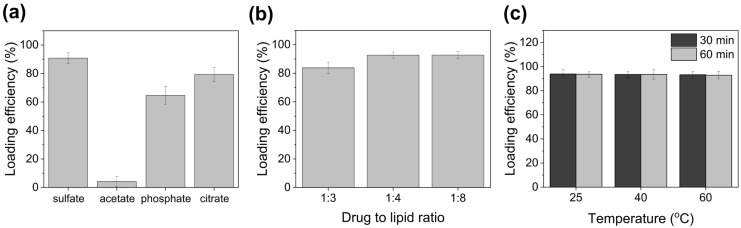
Optimization of EXT encapsulation efficiency in IMP305. Encapsulation efficiency dependent on (**a**) the ionic composition (ammonium sulfate, ammonium acetate, ammonium phosphate, and ammonium citrate), (**b**) the drug-to-lipid ratio (1:3, 1:4, and 1:8, *w*/*w*), and (**c**) temperature (25, 40, and 60 °C) for 30 and 60 min.

To optimize EXT encapsulation into IMP305, encapsulation efficiency was investigated by varying the ionic gradient, drug-to-lipid ratio, and incubation conditions. Figure 3a showed that, among the ammonium-based salts (sulfate, acetate, phosphate, and citrate) at a 250 mM concentration, ammonium sulfate resulted in the highest encapsulation efficiency (90.84 ± 3.88%) owing to a strong transmembrane pH gradient and efficient trapping of the protonated drug, consistent with the classical remote-loading mechanism for weakly basic compounds [28]. Ammonium phosphate and ammonium citrate resulted in moderate encapsulation efficiencies of 64.64 ± 6.22% and 79.43 ± 4.98%, respectively. Specifically, the ammonium acetate gradient failed to sustain a sufficient transmembrane proton and ammonium gradient, resulting in a markedly reduced loading efficiency (4.13 ± 3.54%). The simple acetate gradients leaded to lower efficiencies due to weaker ion-pairing capacity and rapid dissipation of the internal gradient [29].

To determine the optimal drug-to-lipid ratio, the lipid concentration was fixed at 16 mg/mL while varying the drug amount to achieve weight ratios of 1:8, 1:4, and 1:3. Both 1:8 (92.75 ± 3.96%) and 1:4 (92.71 ± 2.15%) ratios maintained high encapsulation efficiencies, but a further increase to 1:3 resulted in a notable decrease (83.85 ± 2.41%), suggesting saturation of the internal aqueous core. The theoretical loading capacity was estimated at approximately 29.84%, supporting the 1:4 ratio as optimal (Figure 3b). Encapsulation efficiency was also assessed under different thermal conditions (25, 40, and 60 °C) and incubation times (30 and 60 min). High encapsulation efficiency, >92.8%, was consistently observed across all conditions (Figure 3c). However, to minimize potential lipid degradation and ensure process reproducibility, 30 °C for 30 min was selected as the optimal loading condition. Collectively, these findings establish 250 mM ammonium sulfate, a drug-to-lipid ratio of 1:4 (*w*/*w*), and an incubation condition of 30 °C for 30 min as the optimized parameters for robust and reproducible formulation of IMP305.

### 3.3. Ultrasound-Triggered Structural Disruption

**Figure 4:**
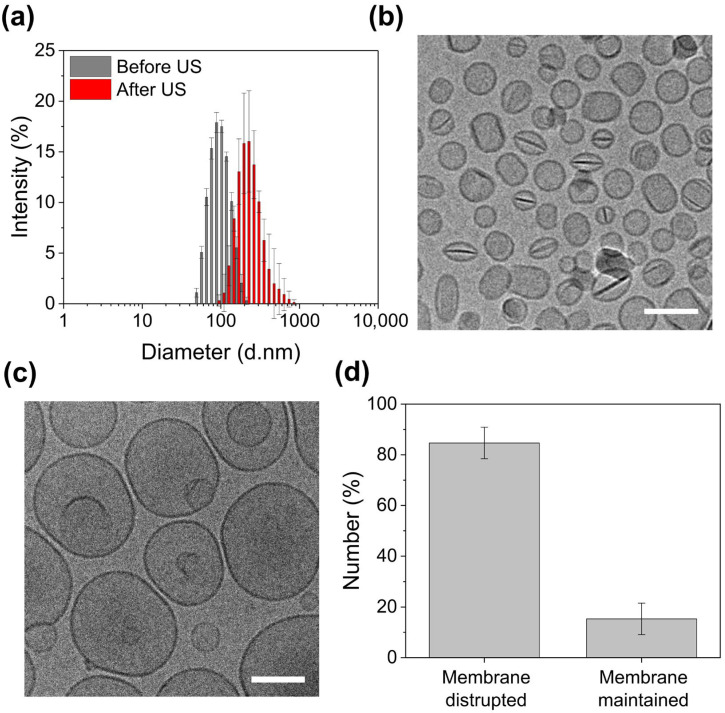
Ultrasound-triggered structural changes of IMP305. (**a**) Size distribution of IMP305 before and after ultrasound (US) exposure, as measured by DLS. Cryo-TEM images of IMP305 (**b**) before and (**c**) after US irradiation. (**d**) Quantification of IMP305 membrane disruption after US exposure. Scale bar: 100 nm.

To evaluate ultrasound responsiveness, IMP305 was exposed to continuous-wave (CW) ultrasound at a 24 kHz frequency and 92 W/cm^2^ for 1 min. The size distribution and morphology of IMP305 were analyzed before and after CW US irradiation. Figure 4a shows an increase in size distribution from 88.5 ± 12.5 nm (PDI: 0.096) to 194.2 ± 39.4 nm (PDI: 0.312). In the results of cryo-TEM images, EXT fiber was observed in the core of IMP305. As mesylate ions were exchanged to sulfate, EXT-sulfate was crystalized with the saturation of sulfate ions as shown in Figure 4b. Upon ultrasound exposure, EXT was released from IMP305, as indicated by the disappearance of EXT fibrous structures, and morphology changed owing to membrane destabilization (Figure 4b,c).

Cryo-TEM images were analyzed for semi-quantification of IMP305 membrane disruption. By evaluating change of size and the disappearance of exatecan crystals in the IMP305 after ultrasound exposure, IMP305 was disrupted at the ratio of 84.68 ± 6.21% (Figure 4d). These findings confirm that IMP305 undergoes structural deformation upon ultrasound exposure, facilitating EXT release.

### 3.4. In Vitro US-Triggered EXT Release from IMP305

**Figure 5:**
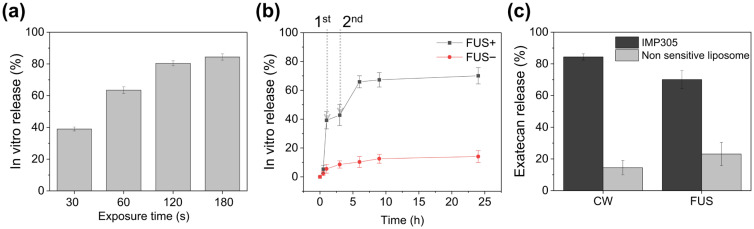
In vitro EXT release profiles from IMP305 under ultrasound stimulation. (**a**) Continuous-wave (CW) ultrasound-induced release with duration of 30, 60, 120, and 180 s. (**b**) Time-dependent release profile with or without FUS exposure with two sequential treatments, demonstrating reproducible ultrasound-triggered drug release. (**c**) EXT release from IMP305 and non-sensitive liposome under CW and FUS irradiation.

The release kinetics of EXT from IMP305 were assessed under both non-focal CW and focused ultrasound (FUS) conditions. CW irradiation at a 24 kHz frequency and 92 W/cm^2^ induced a time-dependent increase in EXT release for evaluation of cavitation-mediated release using a low frequency. In vitro release rates of EXT under the CW irradiation for 30, 60, 120, and 180 s were 39.0 ± 1.23%, 63.5 ± 2.11%, 80.4 ± 1.58%, and 84.4 ± 1.95%, respectively (Figure 5a). In addition, FUS exposure (2.0 kW/cm^2^ intensity, 1% duty cycle, 250 Hz PRF for 10 s/spot) induced robust EXT release (Figure 5b). Prior to FUS irradiation until 0.5 h, the EXT release ratio was only 5.36 ± 2.63%. However, 0.5 h after FUS irradiation, the EXT release ratio was sufficiently increased at the time point of 1 h (39.2 ± 5.98%). Thus, the EXT release ratio was further increased at 6 h after the second FUS irradiation (65.9 ± 4.17%), whereas only 3.6% was released without FUS exposure between 1 h and 3 h (42.8 ± 7.23%). And most of the EXT was released at the time point of 24 h (70.1 ± 5.67%). These results demonstrate that IMP305 enables on-demand, cavitation-triggered EXT release. In contrast, without FUS irradiation, no burst release was observed, with EXT release rates of 5.54 ± 3.02%, 8.54 ± 2.52%, 10.31 ± 3.75%, 12.57 ± 3.07%, and 14.08 ± 4.12% at 1, 3, 6, 9, and 24 h, respectively.

To compare EXT release capacity by ultrasound response, EXT release was quantitatively evaluated in IMP305 and non-sensitive liposomes under CW (92 W/cm^2^, 180 s) and FUS (2.0 kW/cm^2^ intensity, 1% duty cycle, 250 Hz PRF for 10 s/spot) conditions. As shown in Figure 5c, IMP305 exhibited a markedly higher release of 84.4 ± 1.95%, whereas non-sensitive liposomes released only 14.5 ± 4.55% under CW irradiation. Similarly, under FUS exposure, IMP305 achieved a release of 70.1 ± 5.67%, which remained substantially greater than that of the non-sensitive liposomes (23.1 ± 7.31%).

### 3.5. In Vitro Cellular Uptake and Cytotoxicity of IMP305 Under the US Irradiation

**Figure 6:**
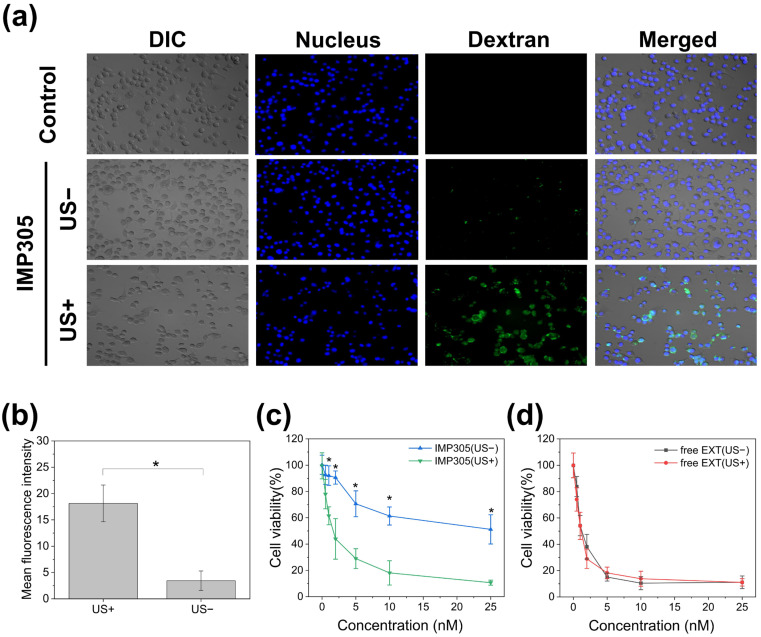
Cellular uptake and cell viability of IMP305 in PANC-1 cells. (**a**) Confocal laser scanning microscopy images of IMP305 with (US+) or without (US−) ultrasound irradiation. Nuclei (blue, Hoechst), FITC–dextran (green). (**b**) Quantified cellular uptake efficiency of FITC–dextran fluorescence intensity with (US+) or without (US−) ultrasound irradiation. Cell viability of (**c**) IMP305 and (**d**) free EXT with or without US exposure. * *p* < 0.05.

Cellular uptake of IMP305 was investigated in PANC-1 cells using FITC-conjugated dextran-4k encapsulated IMP305 for the fluorescent analysis. Figure 6a shows the fluorescence confocal microscopy of cellular uptake. As it is negatively charged (Figure 2b), IMP305 was rarely able to permeate through the PANC-1 cell membrane in the absence of ultrasound (US−), whereas a cytosolic fluorescence signal was observed in the group of ultrasound exposure (US+). These findings indicated that the drug was not released and exhibited no cytotoxicity in the absence of ultrasound exposure, however, the drug was effectively internalized into the cells upon ultrasound-triggered release. Also, quantitative analysis of FITC–dextran fluorescence revealed that the mean fluorescence intensity increased by approximately 5.26-fold upon ultrasound irradiation, rising from 3.45 ± 1.84% in the absence of ultrasound to 18.14 ± 3.50% under ultrasound exposure (Figure 6b).

For the analysis of cell viability with the combination of IMP305 and US irradiation, IMP305 encapsulating EXT was applied to the PANC-1 cells. The concentrations of EXT in IMP305 and free EXT were from 0.5 to 25 nM. The cell viability of IMP305 and free EXT is shown in Figure 6c,d.

The cytotoxicity of IMP305 and free EXT was evaluated with or without ultrasound exposure using the MTS assay. In the results of cell viability, IMP305 exhibited strong US-dependent cytotoxicity as shown in Figure 6c, while free EXT showed cytotoxicity independent of US exposure (Figure 6d). At 25 nM, cell viability of IMP305 was 51.20 ± 11.12% (US–) and 10.67 ± 1.90% (US+), representing an ≈79% reduction upon ultrasound activation. At 10 nM, cell viability decreased from 61.37 ± 6.86% (US−) to 18.10 ± 9.25% (US+), and at 5 nM from 70.67 ± 9.85% (US−) to 28.97 ± 7.55% (US+). In contrast, free EXT indicated similar cytotoxicity regardless of ultrasound exposure: 11.13 ± 4.81% (US−) versus 11.03 ± 2.80% (US+) at 25 nM. Across all concentrations (0.5–25 nM), IMP305 + US exhibited 2–4-fold greater cytotoxicity than IMP305 alone (*p* < 0.05), confirming that ultrasound-triggered release significantly enhanced intracellular drug availability and reduced systemic toxicity before ultrasound irradiation for EXT release.

### 3.6. In Vivo Toxicity Assessment

**Figure 7:**
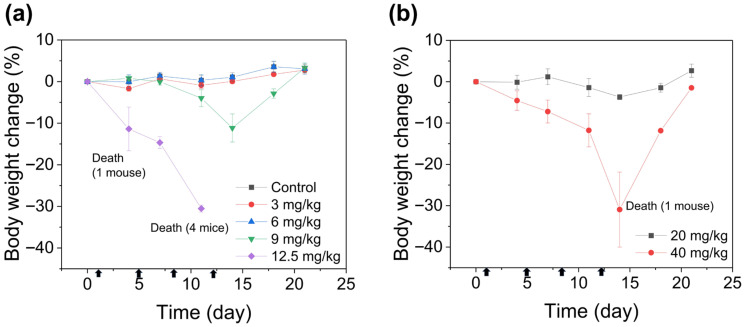
In vivo toxicity assessment. Body weight changes of mice treated with escalating doses of (**a**) free EXT (3, 6, 9, and 12.5 mg/kg) and (**b**) IMP305 (20 and 40 mg/kg for EXT). Arrows indicate the days of free EXT or IMP305 administration.

To assess the reduction of EXT systemic toxicity by encapsulation into IMP305, in vivo tolerability of free EXT and IMP305 was compared in BALB/c nude mice. Figure 7 shows the body weight change after intravenous administration of IMP305 or free EXT. Free-exatecan-treated mice significantly demonstrated dose-dependent body weight loss. In the groups of 3 and 6 mg/kg, the loss of body weight was not observed in four administrations. However, at a dose of 9 mg/kg, the body weight of mice treated with free EXT decreased after the final administration (−11.15 ± 3.38%). The body weight gradually recovered once the administration was discontinued. Thus, a 12.5 mg/kg dose administered to mice showed sufficient loss of body weight. The values of body weight change was –11.37 ± 5.26% and −30.53 ± 0.83% at 11 days and 14 days, respectively. Particularly, all mice (*N* = 5) were dead at the 1st administration (*N* = 1) and the 3rd administration (*N* = 4) in the highest-dose group by acute toxic response. In contrast, IMP305 was tolerated up to 20 mg/kg, with slight body weight loss at the final administration of IMP305 (14 days). Body weight was recovered in the 20 mg/kg group and remained stable through day 21. And at the dose of 40 mg/kg, only one mouse was dead with a −30.9 ± 9.07% (14 day) body weight loss. And body weight was dramatically recovered as administration was stopped. These findings indicate that liposomal encapsulation of exatecan substantially improved tolerability, resulting in an approximately four-fold expansion of the safe dosing range compared with free EXT.

### 3.7. Temperature Simulation During FUS Irradiations

**Figure 8:**
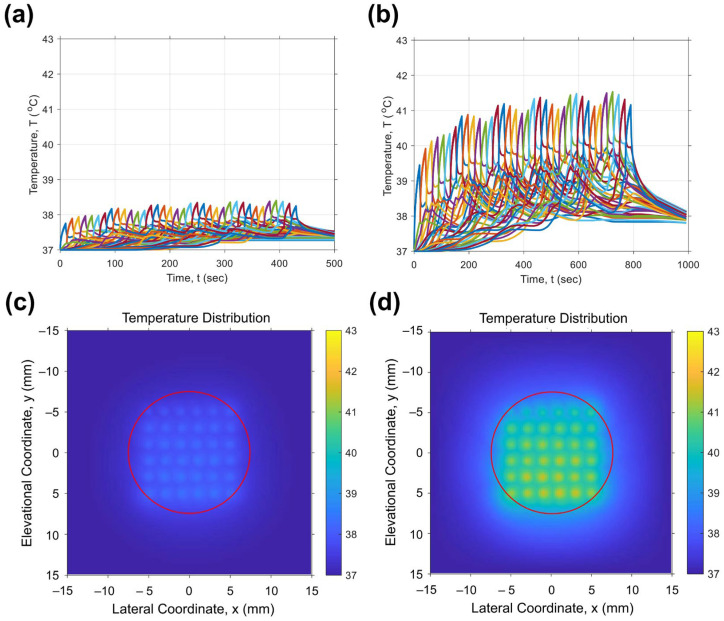
Temperature simulation results during FUS irradiation. Temperature changes at each spots are shown for (**a**) 2.0 kW/cm^2^ with a 1% duty cycle and (**b**) 2.5 kW/cm^2^ with a 2% duty cycle. Color maps of the FUS exposure region are presented for (**c**) 2.0 kW/cm^2^ with a 1% duty cycle and (**d**) 2.5 kW/cm^2^ with a 2% duty cycle. Red circles demonstrated target region.

To investigate the mechanism of exatecan (EXT) release from IMP305, temperature during FUS irradiation was simulated in a tumor-mimicking environment. The tumor was modeled as a 1 cm diameter circle, and the FUS irradiation spots were arranged with 2 × 2 mm spacing. To evaluate the mechanical cavitation or thermal effects generated by FUS, temperature changes at each point were simulated and were shown using different colors. (Figure 8). Under the FUS condition of 2.0 kW/cm^2^ with a 1% duty cycle, temperature was maintained under 39 °C (Figure 8a). This result supports that the EXT was released by FUS-mediated cavitation, as shown in Figure 5c. Upon FUS irradiation with 2.5 kW/cm^2^ with a 2% duty cycle, temperature was slightly increased to a maximal temperature 41.4 °C. While there was an increase in temperature, it was not sufficient to induce apoptosis or necrosis.

In our previous study, cavitation at the tumor site in a PANC-1 xenografted mouse was measured under conditions similar to those used in the present study [19]. The results showed that cavitation was dependent on acoustic intensity and low duty cycle. Furthermore, FUS parameters that primarily induced thermal effects did not generate cavitation, even at high intensity (1.0 kW/cm^2^). Based on the results from Figure 8 and our previous study, cavitation-induced pressure appears to be the dominant mechanism for EXT release from IMP305.

### 3.8. In Vivo Release of Aluminum Phthalocyanine in IMP305

**Figure 9:**
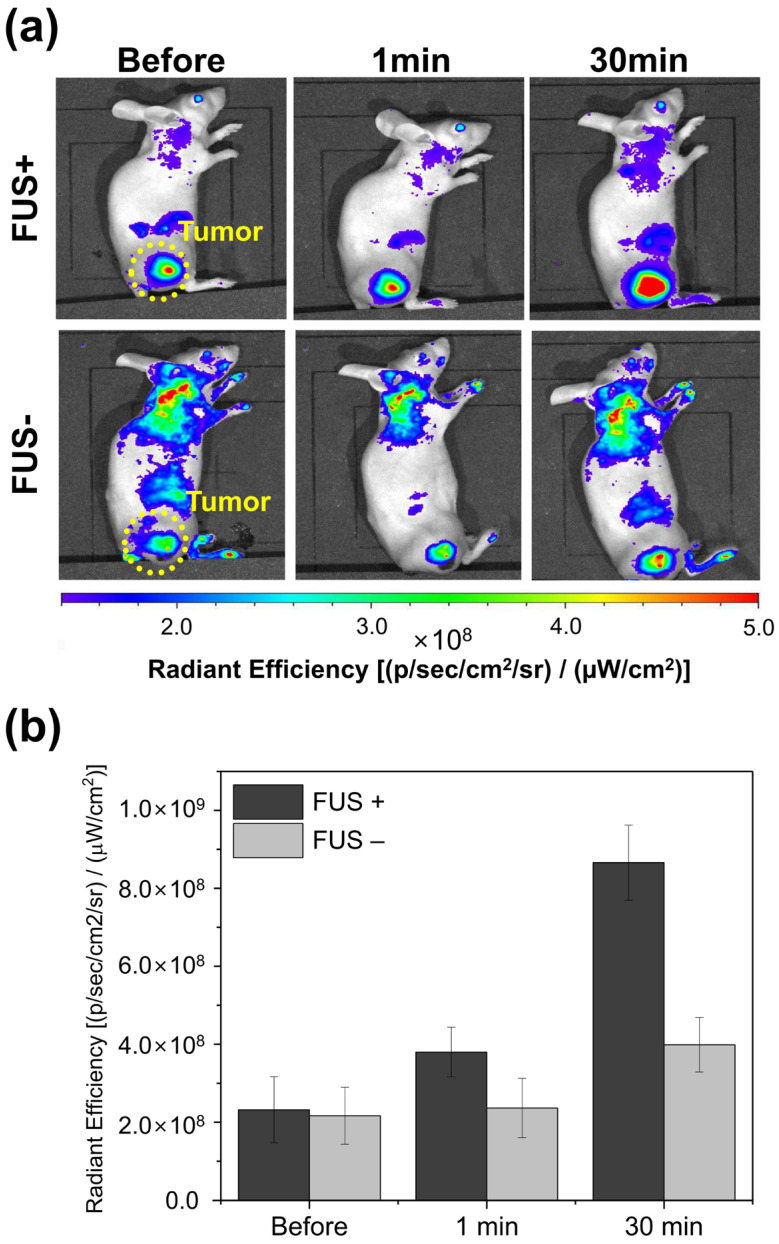
In vivo release capacity of AlPc-encapsulated IMP305 with or without FUS irradiation. (**a**) Representative fluorescence images of PANC-1 xenografted mice after intravenous injection of AlPc-loaded IMP305 at pre-FUS and post-FUS irradiation of 1 min and 30 min. (**b**) Quantitative radiant efficiency analysis at the tumor region before FUS irradiation, 1 min and 30 min after FUS irradiation.

To analyze the FUS-triggered release capacity in IMP305 in the PANC-1 xenografted mouse model, the aluminum phthalocyanine (AlPc)-encapsulated IMP305 was prepared by passive encapsulation. AlPc encapsulated within IMP305 was self-quenched, thereby exhibiting negligible intrinsic fluorescence intensity in an intact state. Upon FUS exposure, AlPc was released from the IMP305, resulting in recovery of the fluorescence intensity. This property was used to evaluate FUS-mediated release in vivo in the tumor region. After intravenous administration of AlPc-encapsulated IMP305, fluorescence imaging of mice was visualized at 670/680 nm (ex/em). Figure 9 demonstrates the fluorescence images with/without FUS exposure. Basically, the fluorescence intensity of the tumor was enhanced by accumulation of IMP305 by an enhanced permeability and retention (EPR) effect. And AlPc was rarely released in the tumor region. Before FUS irradiation, the average radiant efficiency at the tumor site was 2.17 × 10^8^ (p/s/cm^2^/sr)/(mW/cm^2^) in the FUS– group and 2.32 × 10^8^ (p/s/cm^2^/sr)/(mW/cm^2^) in the FUS+ group. The fluorescence intensity was similarly detected in the tumor region without FUS exposure, whereas FUS exposure resulted in a sufficient increase in fluorescence intensity. At 1 min of FUS irradiation, fluorescence intensities increased to 3.80 × 10^8^ ± 0.64 × 10^7^ (FUS+), compared to 2.37 × 10^8^ ± 0.76 × 10^7^ (FUS−). And the fluorescence intensity reached 8.66 × 10^8^ ± 0.47 × 10^8^ 30 min after FUS irradiation (FUS-; 3.99 × 10^8^ ± 0.70 × 10^8^). The fluorescence intensity was gradually increased as AlPc was continuously released in IMP305. Quantitatively, FUS exposure produced a 2.2-fold higher fluorescence signal at 30 min compared with non-irradiated controls (*p* < 0.05). These results indicate that focused ultrasound significantly enhances the tumor accumulation of IMP305 liposomes, confirming effective site-specific drug release.

### 3.9. Antitumor Efficacy with the Combination of IMP305 and FUS Irradiation

**Figure 10:**
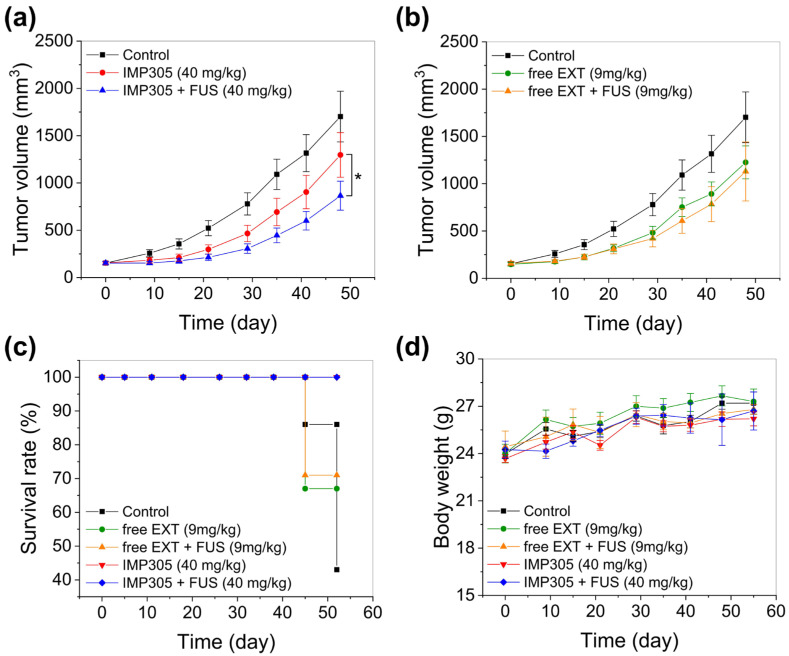
In vivo antitumor efficacy of IMP305 in PANC-1 xenograft-bearing mice. (**a**) Tumor volume growth following treatment with IMP305 and IMP305 + FUS (40 mg/kg for EXT), compared with control. (**b**) Tumor volume growth with the treatment of free EXT and free EXT + FUS (9 mg/kg). (**c**) Survival analysis and (**d**) body weight changes during the treatment period.

Antitumor efficacy was evaluated in PANC-1 xenograft BALB/c nude mice with intravenous administration of free exatecan and IMP305. As analyzed in Section 3.6, the tolerance dose was 9 mg/kg for free EXT and 40 mg/kg for IMP305. Therefore, a maximal tolerance dose of each agent was applied for the antitumor efficacy analysis. Figure 10 shows tumor growth, survival ratio, and body weight for IMP305 and free EXT. Tumor growth in the control group continuously increased, whereas IMP305 and free EXT resulted in effective suppression of tumor growth (Figure 10a,b). Notably, under the FUS exposure, IMP305 resulted in significant reduction in tumor volume compared to IMP305 monotherapy (Figure 10a). On the other hand, suppression of tumor volume was not affected by FUS irradiation in the free EXT group (Figure 10b). On day 48, the tumor volume in the control group reached 1702.05 ± 267.99 mm^3^, compared with 1296.69 ± 236.07 mm^3^ for IMP305 and 865.22 ± 153.20 mm^3^ for IMP305 + FUS, corresponding to tumor growth inhibition (TGI) of 23.82 ± 13.87% and 49.17 ± 9.00%, respectively. Free exatecan (9 mg/kg) yielded 1225.53 ± 174.44 mm^3^ (TGI; 23.82 ± 13.87%), and free exatecan + FUS resulted in 1129.76 ± 311.93 mm^3^ (TGI, 33.62 ± 18.33%).

Corresponding to the antitumor efficacy results, survival analysis further supported that IMP305 showed tumor-specific EXT release by FUS irradiation. The IMP305 + FUS and IMP305 groups maintained 100% survival throughout the entire 50-day period, whereas survival rates declined to 43% in the control, 67% in the free exatecan group, and 71% in the free exatecan + FUS group by the end of the study (Figure 10c). Body weight remained stable (24–28 g) with no significant between-group variation, supporting the absence of systemic toxicity (Figure 10d).

These results collectively demonstrate that FUS-sensitive EXT release of IMP305 achieved marked antitumor efficacy while maintaining systemic safety.

## 4. Conclusions

In this study, we developed a focused ultrasound-sensitive liposomal exatecan mesylate (IMP305) as a strategy to overcome the limitations of free exatecan and to enhance penetration into PDAC by cavitation. IMP305 exhibited a nanoscale diameter with a narrow size distribution, high encapsulation efficiency of exatecan mesylate, and stable colloidal properties, all characteristics critical for systemic delivery. Importantly, IMP305 showed ultrasound-responsive behavior, particularly cavitation, with both continuous-wave and focused ultrasound eliciting rapid and reproducible EXT release. These physicochemical and functional attributes support IMP305 as a rationally designed nanocarrier platform for ultrasound-mediated chemotherapy.

To evaluate the therapeutic relevance of ultrasound-responsive release, IMP305 was compared with free EXT. In vitro, free EXT induced cytotoxicity irrespective of FUS, reflecting immediate drug availability. In contrast, IMP305 induced minimal cytotoxicity without FUS irradiation, indicating that the payload remained within the IMP305. Cytotoxicity was induced under the FUS irradiation, which resulted in rapid EXT release.

These characteristics were reproduced in vivo. Body weight profiles during dose examination demonstrated that IMP305 had an enhancement of tolerability and supported a higher MTD than free exatecan. Cavitation-mediated payload release was also confirmed in an in vivo experiment, as the local fluorescence signal increased only at the FUS-exposed tumor site and with simulation of temperature change during FUS irradiation. Antitumor activity showed a similar profile. Free exatecan exhibited comparable efficacy irrespective of ultrasound exposure. In contrast, IMP305 combined with FUS produced the most effective tumor growth inhibition, indicating that FUS-triggered release of EXT in IMP305 confers superior therapeutic benefit compared with either free drug or IMP305 without FUS exposure.

Overall, IMP305 provides a strategy to overcome the limitations of free exatecan by reducing systemic toxicity and enabling tumor-specific EXT release under FUS irradiation. This strategy may also be applicable beyond pancreatic cancer, including other tumor types and therapeutic payloads that would benefit from local release.

Further study should include detailed pharmacokinetic characterization, refinement of dosing, and FUS treatment protocols and validation in additional tumor models for translation to patients. In conclusion, FUS-sensitive liposomes such as IMP305 may serve as a clinically relevant platform to improve the therapeutic index and are expected to be translated into clinical application.

## Data Availability

Data are contained within the article.
